# The Timing and Extent of Motor Neuron Vulnerability in ALS Correlates with Accumulation of Misfolded SOD1 Protein in the Cortex and in the Spinal Cord

**DOI:** 10.3390/cells9020502

**Published:** 2020-02-22

**Authors:** Baris Genc, Oge Gozutok, Nuran Kocak, P. Hande Ozdinler

**Affiliations:** 1 Ken and Ruth Davee Department of Neurology and Clinical Neurological Sciences, Feinberg School of Medicine Chicago, IL 60611, USA; b-genc@northwestern.edu (B.G.); oge.gozutok@northwestern.edu (O.G.); nuran.kocak@northwestern.edu (N.K.); 2 Chemistry of Life Processes Institute, Northwestern University, Chicago, IL 60201, USA; 3 Les Turner ALS Center, Feinberg School of Medicine Chicago, IL 60611, USA; 4 Mesulam Center for Cognitive Neurology and Alzheimer’s Disease, Northwestern University Feinberg School of Medicine Chicago, IL 60611, USA; 5 Robert H. Lurie Comprehensive Cancer Center, Northwestern University, Chicago, IL 60611, USA

**Keywords:** corticospinal motor neuron, ALS, misfolded SOD1, motor neurons, selective vulnerability

## Abstract

Understanding the cellular and molecular basis of selective vulnerability has been challenging, especially for motor neuron diseases. Developing drugs that improve the health of neurons that display selective vulnerability relies on *in vivo* cell-based models and quantitative readout measures that translate to patient outcome. We initially developed and characterized UCHL1-eGFP mice, in which motor neurons are labeled with eGFP that is stable and long-lasting. By crossing UCHL1-eGFP to amyotrophic lateral sclerosis (ALS) disease models, we generated ALS mouse models with fluorescently labeled motor neurons. Their examination over time began to reveal the cellular basis of selective vulnerability even within the related motor neuron pools. Accumulation of misfolded SOD1 protein both in the corticospinal and spinal motor neurons over time correlated with the timing and extent of degeneration. This further proved simultaneous degeneration of both upper and lower motor neurons, and the requirement to consider both upper and lower motor neuron populations in drug discovery efforts. Demonstration of the direct correlation between misfolded SOD1 accumulation and motor neuron degeneration in both cortex and spinal cord is important for building cell-based assays *in vivo*. Our report sets the stage for shifting focus from mice to diseased neurons for drug discovery efforts, especially for motor neuron diseases.

## 1. Introduction

Amyotrophic lateral sclerosis (ALS) is a motor disease characterized by the loss of upper and lower motor neurons. However, not all motor neurons are vulnerable to degeneration to the same extent in ALS. For example, large fast fatigable (FF) alpha spinal motor neurons (SMN) are the most vulnerable and the small slow-twitch fatigue-resistant (S) alpha SMN and gamma SMN that innervate the intrafusal fibers are more resistant to degeneration [1,2,3,4,5,6,7]. Likewise, not all corticospinal motor neurons (CSMN) degenerate to the same extent even at the end-stage. The reason for this varying degree of vulnerability and resistance to degeneration is not fully understood. Misfolded proteins are a hallmark of neurodegenerative diseases [8,9] including ALS [10,11]. Toxic gain of function of mutated misfolded SOD1 protein has been one of the most-widely studied underlying causes of ALS [7], but even today we do not know why different motor neuron pools display a wide range of degeneration in disease. Understanding the basis of selective vulnerability even within motor neuron pools would be instrumental for building treatment strategies. In addition, it can be used as criteria for an outcome measure in drug discovery studies, which are designed to monitor motor neuron survival upon compound treatment. Therefore, revealing a potential correlation between misfolded SOD1 accumulation and motor neuron degeneration, both in the cortex and in the spinal cord, is an important task that has not yet been fully addressed.

Misfolded SOD1 protein can be detected in the SMN in the ventral horn of the hSOD1^G93A^ mouse spinal cord as early as postnatal day seven (P7) [12,13,14,15]. Several misfolded SOD1 antibodies such as A5C3, B8H10, and D3H5, show that misfolded SOD1 colocalizes with choline acetyltransferase positive (ChAT+) SMN [16], allowing investigation, monitoring, and quantitative assessment of SMN that have misfolded SOD1 accumulated in their cytoplasm. In addition, misfolded SOD1 is restricted to large cell bodies of SMN between P30 and P85, after which intense punctate mSOD1 aggregates localized in contiguous processes and in the neuropil throughout the spinal cord with very few discernable cell bodies as the disease progresses [12].

CSMN degenerate together with SMN in ALS, and CSMN are crucial for the initiation and modulation of voluntary movement. Investigating CSMN is challenging mostly because they are very few in numbers and they are embedded within the complex and heterogeneous structure of the motor cortex [17,18]. However, effective and long-term treatment strategies need to incorporate strategies that monitor and assess their response to treatment. Previous studies have shown that in the primary motor cortex, about 40% of layer 5 neurons were reported to contain misfolded SOD1 even at P20 [19]. It is thus important to investigate the presence of a potential correlation between misfolded SOD1 accumulation and CSMN vulnerability and degeneration, as this information can be utilized to assess the potency of compound treatments, especially for upper motor neurons in ALS and other related diseases.

In an effort to visualize motor neurons, we recently generated UCHL1-eGFP reporter mice, in which CSMN in the motor cortex and a subset of SMN in the spinal cord are genetically labeled with eGFP expression that is stable and long-lasting [20]. When hSOD1^G93A^ mice—the golden standard for ALS drug discovery efforts for the past 15 years—are crossed with UCHL1-eGFP reporter mice to generate hSOD1^G93A^-UeGFP ALS reporter mouse model, a significant reduction in the number CSMN was observed, but in the spinal cord, eGFP expression was restricted mostly to S and gamma SMN resistant to degeneration in ALS for unknown reasons [20].

In this study, we investigate the presence of a direct correlation between misfolded SOD1 accumulation and motor neuron vulnerability in both cortex and spinal cord throughout the disease. This information reveals one of the underlying causes of selective vulnerability even within the same motor neuron pools. Most importantly, it also lays a foundation for future drug discovery efforts, which utilize improved motor neuron survival as an outcome measure.

## 2. Materials and Methods

### 2.1. Mice

All animal procedures were approved by the Northwestern University Animal Care and Use committee on November 1st 2018 (Protocol: IS00009980) and comply with the standards of the National Institutes of Health. Northwestern University has an Animal Welfare Assurance on file with the Office of Laboratory Animal Welfare (A3283-01). Transgenic hemizygous males expressing high copy number of the human *SOD1* gene with the G93A mutation (B6SJL-Tg(SOD1*G93A)1Gur/J; The Jackson Laboratory) were bred to hemizygous UCHL1-eGFP (C57BL/6-Tg(Uchl1-EGFP)G1Phoz/J; The Jackson Laboratory) females to generate hSOD1^G93A^-UeGFP and WT-UeGFP (control) mice as described previously [20]. Transgenic mice were identified by PCR amplification of DNA extracted from tail as previously described [20,21]. In this study, WT-UeGFP (*n* = 11) and hSOD1^G93A^-UeGFP (*n* = 15) mice of either sex were used. Mice at P30 (presymptomatic), P60 (early symptomatic), P90 (symptomatic), and P140 (end-stage) were used for analysis. All mice were on the C57BL/6 background.

### 2.2. Histology

Adult mice were deeply anesthetized using ketamine (90 mg/kg) with xylazine (10 mg/kg), and transcardially perfused with 4% PFA in PBS. The brains and spinal cords were removed intact and post-fixed (4% PFA, overnight) and stored in PBS with sodium azide (0.01%) at 4 °C. Sections were cut in coronal (50 μm) planes using a vibratome (Leica, Buffalo Grove, IL, USA) and collected in 6-well plates.

### 2.3. Immunocytochemistry and Cellular Staining

Antibodies used are as follows: anti-misfolded SOD1 (1:200, Clone B8H10, MediMabs; Montreal, Quebec, Canada), anti-GFP (1:500, Thermo Scientific, Waltham, MA, USA). Briefly, sections were treated with blocking solution (PBS, 0.05% BSA, 2% FBS, 1% Triton X-100, and 0.1% saponin) for 30 min followed by incubation with primary antibody solution diluted in blocking solution overnight at 4 °C. Appropriate secondary fluorescent antibodies (1:500, AlexaFluor-488 or AlexaFluor-647 conjugated, Invitrogen, Waltham, MA, USA) were added to the blocking solution at room temperature for 2 h in the dark. All samples were subjected to the immunofluorescent staining at the same time using the same antibody cocktail.

### 2.4. Imaging

Low-magnification images were acquired using an Eclipse TE2000-E (Nikon Instruments Inc., Melville, NY, USA) for qualitative analysis of GFP expression patterns at different ages. An LSM880 (Carl Zeiss Microscopy LLC., White Plains, NY, USA) was used to image and collect Z-stack images of the motor cortex at P30, P60, P90, and P140. Confocal images were captured on multiple sessions with the same pinhole and laser settings so that image intensities would be the same for each image. Z-stacks were processed to generate maximum intensity projections.

### 2.5. Data Collection and Analysis

The number of CSMN that included misfolded SOD1 (eGFP+ and B8H10+ neurons) in the 25X oil field of view of primary motor cortex of hSOD1^G93A^-UeGFP mice were quantified (*n* = 94 to 161 neurons per mouse, P30: *n* = 3 mice; P60: *n* = 4 mice; P90: *n* = 4 mice, and P140: *n* = 4 mice). The average percentage of CSMN that included misfolded SOD1 was determined based on these quantifications. In WT mice, none of the CSMN included misfolded SOD1. Likewise, the number of cells that are not CSMN [eGFP negative (eGFP-)] and that contain misfolded SOD1 (B8H10+) were also quantified in layer 5 of the motor cortex. The average percentage of the non-CSMN cells that contain misfolded SOD1 protein was reported; the mean and standard error of mean (S.E.M.) was also determined. Please refer to Table 1.

Statistical analysis was performed using Prism (GraphPad, San Diego, CA, USA). Ordinary one-way ANOVA with Tukey’s multiple comparisons test was used to determine adjusted *p* values.

## 3. Results

### 3.1. Misfolded SOD1 Is Expressed mainly in Layer 5 of the Motor Cortex and Colocalizes with Diseased CSMN

**Figure 1:**
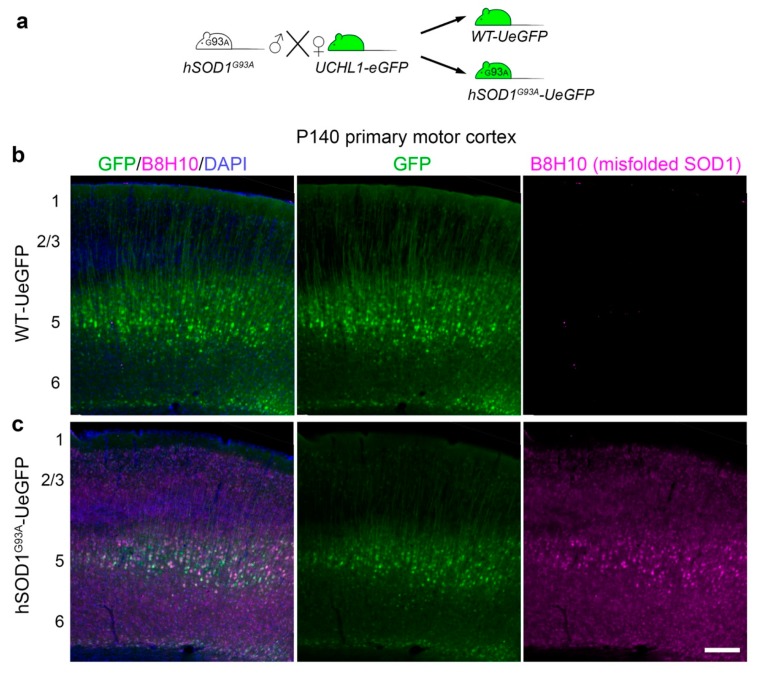
Generation of hSOD1^G93A^-UeGFP amyotrophic lateral sclerosis (ALS) reporter mouse model and detection of misfolded SOD1 protein. (**a**) hSOD1^G93A^-UeGFP mice were generated by breeding hSOD1^G93A^ male mice carrying high copy number of human SOD1 protein with a point mutation at position 93 with female UCHL1-eGFP reporter mice that label corticospinal motor neurons (CSMN) with eGFP expression; (**b**,**c**) B8H10 antibody detects misfolded human SOD1 protein in the primary motor cortex of hSOD1^G93A^-UeGFP (**c**) but not WT-UeGFP (**b**) mice. Misfolded SOD1 signal is the brightest in layer 5 where GFP+ CSMN are located. Scale bar, 250 μm.

hSOD1^G93A^-UeGFP mice were previously generated by crossing hSOD1^G93A^ with UCHL1-eGFP mice (Figure 1a) after CSMN identity of eGFP+ neurons in layer 5 of the motor cortex were confirmed and the numbers of GFP+ CSMN were significantly reduced with disease progression starting at P90 [20]. CSMN in hSOD1^G93A^-UeGFP mice recapitulated the progressive degeneration observed in the hSOD1^G93A^ mice. However, it was not clear whether the accumulation of misfolded SOD1 contributed to CSMN death. CSMN are identified by their expression of eGFP (Figure 1b,c). Their soma is located in layer 5 of the motor cortex and apical dendrite project to the top layers. Misfolded SOD1 antibody B8H10 is specific for labeling not all forms of SOD1, but only the misfolded form [12,13,16,22], allowing us to assess the correlation between the presence of misfolded SOD1 and motor neuron loss in the cortex and the spinal cord. There was no B8H10 signal, and thus no misfolded SOD1 protein in the brains of WT-UeGFP control mice at any age investigated in this study (Figure 1b and Figure S1). In striking contrast, misfolded SOD1 was present in the cortex, the brightest signal observed especially in layer 5, and most co-localized with diseased CSMN of hSOD1^G93A^-UeGFP mice (Figure 1c).

**Figure 2:**
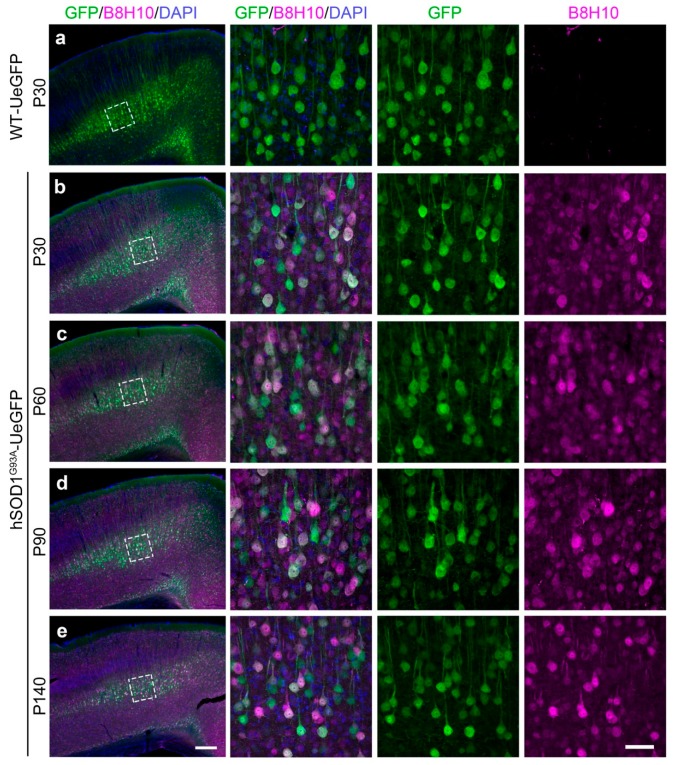
Misfolded SOD1 protein in the primary motor cortex. (**a**) B8H10 antibody does not detect any misfolded SOD1 protein in the primary motor cortex of WT-UeGFP control mice; (**b**–**e**) misfolded SOD1 protein can be detected in the primary motor cortex of hSOD1^G93A^-UeGFP mice by the B8H10 antibody at P30 (**b**), P60 (**c**), P90 (**d**), and P140 (**e**). Boxed areas enlarged in the right panels. Scale bar, 250 μm (left, low mag) and 50 μm (right, high mag).

In an effort to investigate whether diseased CSMN express misfolded SOD1, we studied the motor cortex of hSOD1^G93A^-UeGFP mice at P30, P60, P90, and P140. In WT-UeGFP mice, misfolded SOD1 was not detected (Figure 2a). However, misfolded SOD1 protein was present in eGFP+ CSMN as early as P30 (Figure 2b) before disease onset, and continued to be present at P60 (Figure 2c; early symptomatic), P90 (Figure 2d; symptomatic), and at P140 (Figure 2e; end-stage). Higher magnification and confocal imaging confirmed that low levels of misfolded SOD1 were also present in other cells that are not CSMN (eGFP-; P30: 64.68% ± 2.98%; P60: 58.81% ± 1.1%; P90: 60.08% ± 2.36%; P140 52.41% ± 2.40%). However, most CSMN soma included comparable and high levels of misfolded SOD1 at all ages investigated without statistical significance among ages (P30: 78.52% ± 5.40%; P60: 90.29% ± 1.05%; P90: 86.06% ± 2.74%; P140: 86.37% ± 6.74%), and the intensity of expression increased with disease severity (Table 1).

### 3.2. Misfolded SOD1 Protein Is Detected Primarily in Vulnerable and Degenerating SMN

**Figure 3:**
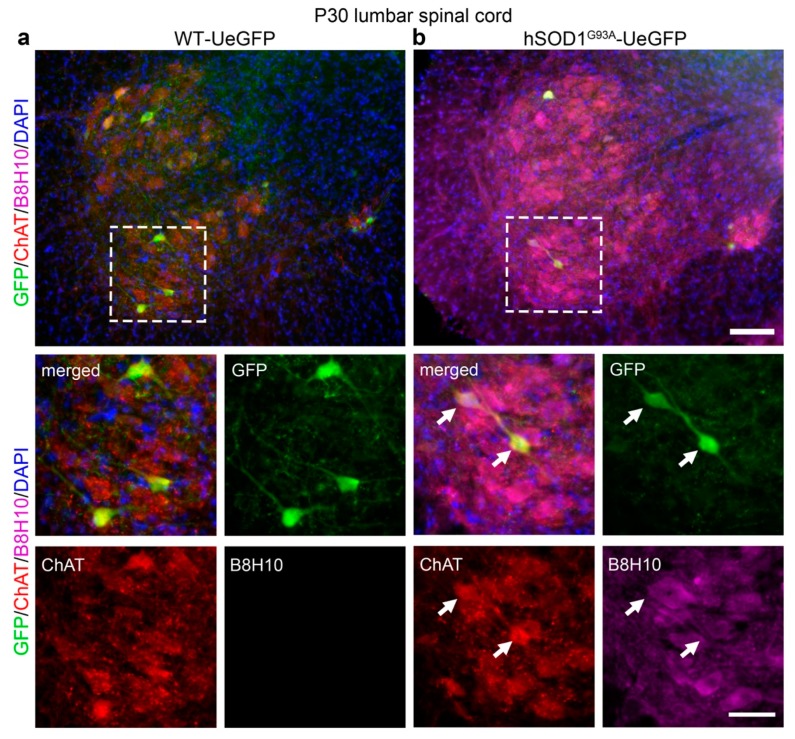
Misfolded SOD1 protein in the ventral horn of the lumbar spinal cord. (**a**) No B8H10 signal is detected in the spinal cord of WT-UeGFP control mice; (**b**) misfolded SOD1 protein can be detected in the lumbar spinal cord of hSOD1^G93A^-UeGFP mice by the B8H10 antibody in ChAT+ SMN. Arrows point to eGFP+ ChAT+ SMN without a misfolded SOD1 signal. Boxed areas enlarged in the panels below. Scale bar, 100 μm (top, low mag) and 50 μm (bottom, high mag).

In the spinal cord of UCHL1-eGFP mice, initially, all SMN are labeled by eGFP expression, but by P30, the expression becomes restricted to a subset of small diameter ChAT+ SMN that are more resistant to neurodegeneration [20]. The lack of eGFP expression in the large diameter SMN that are selectively more vulnerable to degeneration in *hSOD1^G93A^*-UeGFP mice was puzzling [20]. In an effort to investigate a potential correlation between the presence of misfolded SOD1, eGFP expression and vulnerability state of SMN, we investigated the presence of misfolded SOD1 in the SMN of *hSOD1^G93A^*-UeGFP mice at P30, P60, P90, and P140. Misfolded SOD1 was not detected in the spinal cords of WT-UeGFP mice between P30 and P140 (Figure 3a and Figure S2). However, ChAT+ SMN in the ventral horn of *hSOD1^G93A^*-UeGFP mice included misfolded SOD1 as early as P30 (Figure 3b). Interestingly GFP+ SMN were immunopositive for ChAT, but not misfolded SOD1 protein (Figure 3b arrows).

**Figure 4:**
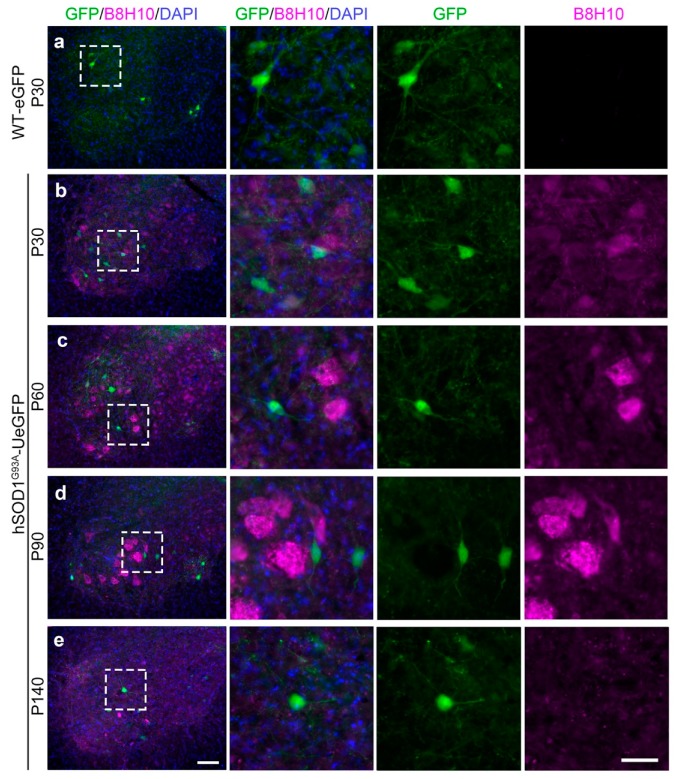
Misfolded SOD1 protein in the ventral horn of the lumbar spinal cord. (**a**) There is no misfolded SOD1 protein in the spinal cord of WT-UeGFP control mice; (**b**–**e**) misfolded SOD1 protein are detected in the lumbar spinal cord of hSOD1^G93A^-UeGFP mice by the B8H10 antibody and degeneration resistant eGFP+ SMN do not have misfolded SOD1 at P30 (**b**), P60 (**c**), P90 (**d**), and P140 (**e**). Boxed areas enlarged in the right panels. Scale bar, 100 μm (left, low mag) and 50 μm (right, high mag).

The detailed analyses over time (Figure 4) further revealed that misfolded SOD1 was not present in all neurons or cells at the same level, and its intensity increased with disease progression especially in the large alpha SMN that are most vulnerable to degeneration. The misfolded SOD1 was not detected in the spinal cord of WT-eGFP mice (Figure 4a), but low levels of expression became present as early as P30 in the spinal cord of *hSOD1^G93A^*-UeGFP mice (Figure 4b). As the disease progressed with age, the intensity of misfolded SOD1 expression increased mainly in the large size SMN, which among all other SMN, become vulnerable very early and display the fastest rate of degeneration [1,2,3,4,5,6,7]. In contrast, the eGFP+ SMN in the *hSOD1^G93A^*-UeGFP mice, which are degeneration resistant, were devoid of misfolded SOD1 at P30 (Figure 4b), P60 (Figure 4c), P90 (Figure 4d), and even at P140 (Figure 4e).

## 4. Discussion

ALS is characterized by selective vulnerability and degeneration of motor neurons both in the cortex and spinal cord [23,24,25,26]. In the brain, the upper motor neurons, which are called Betz cells in patients and CSMN in mice, display selective vulnerability and progressive degeneration [20,27]. Interestingly, in the spinal cord, not all SMN display equal vulnerability and some remain resistant to degeneration. There appears to be a progressive line of degeneration among different subsets of SMN, where S alpha SMN displays initial signs of degeneration, followed by FR and FF remain mostly resistant [20,28,29,30,31,32]. Differentiating vulnerable and degeneration-resistant neurons in a reporter line is of great importance to monitor and assess their responses to compound treatment strategies. It is also important to understand why some motor neurons are more resistant to degeneration than others. This information can be used to modulate other degeneration prone neurons so that they also become resistant.

Mutant SOD1 protein was previously shown to form a misfolded form that has the gain of toxic function, leading to selective degeneration especially in motor neurons. However, even though the mutant SOD1 gene is expressed in all cells and neurons, it was not clear why motor neurons display primary vulnerability. Even though the differential vulnerability of different subtypes of SMN to degeneration in motor neuron diseases is well established [1,2,3,4,5,6,7], the molecular mechanisms underlying the basis of selective vulnerability is beginning to emerge. Therefore, being able to detect misfolded proteins gain attention.

There are numerous antibodies available that recognize various epitopes of the SOD1 protein [33,34]. The antibody used in this study, mouse monoclonal B8H10 antibody, recognizes the exon3 of the SOD1 protein encoding the loop IV in the 3D structure [34,35], and does not immunoprecipitate WT SOD1 protein [36]. In the spinal cord of hSOD1^G93A^ mice, B8H10 antibody shows a similar pattern compared to A5C3 and D3H5 antibodies with misfolded SOD1 protein being detected in motor neurons between P30 and P85, after which all three antibodies detect punctate structures within the neuropil in addition to SMN, and at the end-stage of the disease, only punctate aggregates are detectable throughout the spinal cord [12]. Our findings in the spinal cord using the B8H10 antibody is in agreement with this and other reports of misfolded SOD1 protein accumulation in SMN [12,13,14,15,16]. There has been only one report of misfolded SOD1 protein accumulation in layer 5 neurons in the cortex so far using the same antibody as in our study [19]. Based on the similarity of staining pattern in the spinal cord using different antibodies, it is expected to observe a similar pattern in the motor cortex with different commercially available antibodies as well, but it would be interesting to see if antibodies with differential selectivity for different isotypes of SOD1 protein might yield novel insights into selective vulnerability of CSMN in ALS mouse models.

There have been reports of misfolded SOD1 pathology detected in sporadic ALS cases using conformation-specific antibodies selective for misfolded SOD1 protein species [37], as well as the absence of misfolded SOD1 in sporadic ALS [38]. In light of the prion-like properties of SOD1 protein [11], the potential contribution of misfolded WT SOD1 protein to ALS pathogenesis and neurotoxicity becomes an interesting topic [36,39]. Both the loss of native SOD1 post-translational modifications and the introduction of aberrant post-translational modifications can induce misfolding of WT SOD1 protein, which can be toxic and mimic the fALS-linked mutant SOD1 [36].

Misfolded SOD1 protein has been detected in a subpopulation of alpha SMN at P7 but not earlier in the hSOD1^G93A^ mouse spinal cord [13]. At P60, ~50% of SMN had misfolded SOD1, which were identified as FF alpha SMN by retrograde labeling [13]. By P90, misfolded SOD1 was detected in ~80% of SMN, including both FF and FR alpha SMN subtypes [40]. In hSOD1^G93A^ mice, larger vulnerable SMN undergo a reduction in dendritic length and dendritic spine loss starting as early as P30 before disease onset [41]. Enhancing SMN excitability reduced the misfolded SOD1 load and provided neuroprotection, whereas reducing excitability augmented SOD1 misfolding and accelerated disease [13]. Coimmunoprecipitation experiments using the B8H10 antibody identified Na^+^/K^+^ATPase-α3 as a binding partner of misfolded but not WT SOD1 protein, reducing its activity and modulating the excitability of vulnerable FF SMN [42]. Recently, E3 ubiquitin ligase TRAF6 has been identified as another binding partner of misfolded but not WT SOD1, which might underlie the accumulation of misfolded SOD1 protein in vulnerable neurons [43].

Vulnerable SMN were shown to be selectively prone to ER stress [3]. An ER chaperone SIL1 which highly expressed disease-resistant SMN, but is selectively reduced in vulnerable FF SMN [44]. Loss of Sil1 in SMN with misfolded SOD1 accumulation makes them vulnerable to neurodegeneration [44]. A calcium-binding ER chaperone calreticulin is reduced in SMN vulnerable to ALS [45], and further reduction of calreticulin levels by breeding hSOD1^G93A^ mice with hemizygous calreticulin mutant mice accelerated disease onset and progression [40]. Expression of *mir 17~92* was reduced in vulnerable SMN prior to disease onset in hSOD1^G93A^ mice, and gene therapy using AAV-mediated intrathecal *mir 17~92* delivery improved motor function and survival [46]. Misfolded SOD1 protein binds to the cytoplasmic surface of mitochondria in SMN, and disrupts the normal mitochondria size, shape, and distribution [47]. Enhancing mitochondrial calcium buffering capacity by deleting cyclophilin D, a key regulator of the opening of the mitochondrial permeability transition pore, reduced misfolded SOD1 levels by 80% in the symptomatic stage of hSOD1^G93A^ mouse spinal cord [48]. Failed homeostasis theory of mitochondrial function was previously suggested and dysregulation of respiration, oxidation, and calcium balance could indeed be one of the key contributors to selective vulnerability [49]. In summary, even though we consider SMN as one neuron pool, it contains many different kinds of SMN with different targets, firing potentials, activities, and even molecular signature. So, it should not be surprising that they display different levels of vulnerability in diseases. It is interesting that some SMN do not have misfolded SOD1 and those survive longer. Some SMN have very high levels of misfolded SOD1 and they seem to be affected very early in the disease.

Delivery of SPD1 shRNA using AAV vectors in hSOD1^G37R^ mice reduces levels of misfolded SOD1 protein in the ventral horn of the lumbar spinal cord by ~90%, rescues the alpha SMN numbers and motor function [22]. Matrix metalloproteinase MMP-9 is selectively expressed in fast SMN [50], whereas extracellular matrix protein Osteopontin is selectively expressed in ALS-resistant SMN [51]. Similarly, several strategies are now underway to silence the expression of mutant SOD1 as a therapeutic strategy [22,52,53,54,55,56,57,58,59,60,61,62]. Based on our findings, there is indeed a direct correlation between the presence of misfolded SOD1 and neuronal vulnerability in the spinal cord. The eGFP+ SMN that were degeneration resistant were the ones that lacked misfolded SOD1 expression, and other especially large alpha motor neurons, which displayed very high levels of misfolded SOD1 were the most vulnerable. Therefore, reducing the levels of misfolded SOD1 could indeed be a therapeutic target for the spinal motor neurons.

However, for the upper motor neurons in the motor cortex, the presence of misfolded SOD1 was evident as early as P30 and was persistent until the end-stage. Interestingly the percent average of CSMN that included misfolded SOD1 was relatively comparable throughout. This could be because the percentage of CSMN that degenerate could be similar to the percentage of CSMN that begin to accumulate misfolded SOD1, and thus the overall percentage of CSMN with misfolded SOD1 protein may appear stable at different disease stages. It is important to note that misfolded SOD1 expression was primarily restricted to layer 5, and was not detected in other layers of the motor cortex at any time point. Interestingly, some non-CSMN neurons in layer 5 of the motor cortex also expressed misfolded SOD1, albeit their percentages were much lower than that of CSMN. Therefore, reducing misfolded SOD1 in the cortex would also be beneficial for CSMN and potentially for the overall motor neuron circuitry. Being able to detect differences as early as P30 and having almost stable levels throughout offers great advantages for investigating the impact of compound treatment within a wider window of opportunity *in vivo.*

As the drug discovery efforts are accelerating for motor neuron diseases, including ALS, the need for a reporter line that labels degeneration-resistant SMN is of great importance because the overall goal of compound treatment is to investigate whether the SMN become resistant to degeneration and whether their overall numbers will remain constant or increase. We have previously shown that UCHL1-eGFP reporter mice label a subset of small diameter SMN resistant to neurodegeneration in the *hSOD1^G93A^*-UeGFP ALS reporter mouse model [20]. Now, we show that after disease onset, misfolded SOD1 protein highly and selectively accumulates in large diameter SMN most vulnerable to neurodegeneration, whereas misfolded SOD1 protein seems to be excluded from the ALS-resistant GFP^+^ SMN in *hSOD1^G93A^*-UeGFP ALS reporter mouse model. This finding not only reveals why CSMN and large alpha motor neurons display vulnerability in ALS, but also allows the development of an *in vivo* platform for the assessment of compound treatment in ALS, and other related motor neuron diseases. If the compounds are effective in protecting motor neurons from degeneration, they will continue to express eGFP, allowing quantitative assessment of compound treatment on motor neuron survival. This has been a major disconnect between compound treatment and the improvement of neuron health. In its absence, the motor behavior of mice was used as an outcome measure, which did not translate well in clinical trials, as more than 30 clinical trials failed even though they showed improved behavior in ALS mouse models. Therefore, being able to visualize motor neurons both in the cortex and in the spinal cord of the same reporter line for the disease, enable direct cellular assessment for the efficacy of compound treatment.

## 5. Conclusions

These are exciting times for drug discovery efforts for ALS and other motor neuron diseases. To expedite clinical trials and to increase their success rates, we need to build and characterize tools that can be utilized to assess motor neuron survival upon compound treatment at a cellular level both in the cortex and in the spinal cord. Having both CSMN and SMN labeled in the same reporter line, for the first time, enables investigation of both the upper and the lower motor neuron survival simultaneously. Here, we show that the SMN which do not have misfolded SOD1 remain resistant to degeneration and are eGFP+ in the spinal cord, and this offers a unique opportunity especially for drug discovery efforts, as the overall goal is to reduce toxicity and increase the numbers of neurons that are resistant to degeneration.

## Figures and Tables

**Table 1:** Quantification of neurons containing misfolded SOD1 in primary motor cortex.

Age	Number of Mice	Total Number of Neurons	Average % of Non-CSMN with Misfolded SOD1	S.E.M.	Average % of CSMN with Misfolded SOD1	S.E.M.
P30	3	452	64.68%	2.98%	78.52%	5.40%
P60	4	485	58.81%	1.10%	90.29%	1.05%
P90	4	579	60.08%	2.36%	86.06%	2.74%
P140	4	575	52.41%	2.40%	86.37%	6.74%

## Supplementary Materials

The following are available online at https://www.mdpi.com/2073-4409/9/2/502/s1, Figure S1: Misfolded SOD1 protein is not detected in the cortex of WT-UeGFP reporter mouse, Figure S2: Misfolded SOD1 protein is not detected in the spinal cord of WT-UeGFP reporter mouse.
